# ﻿Taxonomy and phylogeny of *Panus* (Polyporales, Panaceae) in China and its relationship with allies

**DOI:** 10.3897/mycokeys.105.121025

**Published:** 2024-05-31

**Authors:** Lei Yue, Junliang Chen, Yonglan Tuo, Zhengxiang Qi, Yajie Liu, Xiao Lan He, Bo Zhang, Jiajun Hu, Yu Li

**Affiliations:** 1 Engineering Research Centre of Edible and Medicinal Fungi, Ministry of Education, Jilin Agricultural University, Changchun City, 130118, Jilin Province, China; 2 College of Plant Protection, Jilin Agricultural University, Changchun City, 130118, Jilin Province, China; 3 Science and Research Center for Edible Fungi of Qingyuan County, Lishui City, 323800, Zhejiang Province, China; 4 Joint Laboratory of International Cooperation in Modern Agricultural Technology, Ministry of Education, Jilin Agricultural University, Changchun City, 130118, Jilin Province, China; 5 Sichuan Institute of Edible Fungi, Chengdu City, 610066, Sichuan Province, China; 6 School of life science, Zhejiang Normal University, Jinhua City, 321004, Zhejiang Province, China

**Keywords:** Hyphal system, novel species, *Panussimilis*, phylogenetic

## Abstract

*Panus* is a typical wood-rotting fungi, which plays considerable roles in ecosystems and has significant economic value. The genus *Panus* currently consists of more than 100 species; however, only eight species have been reported from China. This study aims to distinguish and describe two novel species from the *Panussimilis* complex, namely *Panusminisporus* and *Panusbaishanzuensis*, one new record species from Zhejiang Province, *Panussimilis* and three common species, *Panusconchatus*, *Panusneostrigosus* and *Panusrudis*, based on detailed morphological and phylogenetic studies, relying on Chinese specimens. *Panusminisporus* is characterised by its reddish-brown pileus, decurrent lamellae with cross-veins, slender stipe, smaller basidiospores, wider generative hyphae and absence of sclerocystidia. *Panusbaishanzuensis* is featured by its pileus with concentric and darker ring zone, decurrent lamellae with cross-veins, shorter stipe, longer basidiospores, diverse and shorter cheilocystidia and smaller sclerocystidia. Internal transcribed spacer (ITS) regions, large subunit nuclear ribosomal RNA gene (nLSU) and translation elongation factor 1-α gene (*tef-1α*) were employed to perform a thorough phylogenetic analysis for genus *Panus* and related genera, using Bayesian Inference and Maximum Likelihood analysis. The results indicate that *Panusminisporus* and *Panusbaishanzuensis* form two independent clades within the *Panussimilis* complex themselves. Detailed descriptions, taxonomic notes, illustrations etc. were provided. In addition, a key to the reported species of *Panus* from China is also provided.

## ﻿Introduction

The genus *Panus* Fr. is one of the wood-rotting fungi with significant economic and ecological value. Its taxonomic history has been carried out for a long time. In 1787, Bulliard (1786) described a new species *Agaricusconchatus* Bull (≡*Panusconchatus* (Bull.) Fr.). When the genus *Panus* was established, it was designated as the type species (Fries 1838). From then on, a large number of new taxa were described around the world (Berkeley and Broome 1883; Stevenson 1964; Senthilarasu 2015; Tibpromma et al. 2017). Till now, there are currently described 113 taxa of *Panus*, including 101 species, 10 varieties, and two metamorphs, according to Index Fungorum (http://www.indexfungorum.org). However, these species were mainly reported from Europe and North America, rarely described from Asia.

The taxonomic status between *Panus* and allies was often disputed, leading to confusion amongst these genera. The key characteristics to separate the genus *Lentinus* Fr. and *Panus* were hugely different in the history research, thus, leading to indistinct boundaries between these genera. Hymenophoral trama structures were adopted by Singer (1961a, 1961b, 1962) and Kühner (1980) to separate *Panus* and *Lentinus*. However, Pegler (1971, 1975) and Corner (1981) employed the hyphal systems as the key features to separate these two genera. These led to confusing and unstable taxonomic systems. Later, hyphal systems were proved to be the more reasonable key characteristics in distinguishing these species (Kibby et al. 1978; Corner 1981; Singer 1986) and treated *Lentinus* and *Panus* as distinctly separate genera. However, Pegler (1983) disagreed that *Panus* was an independent genus and treated it as a subgenus of *Lentinus*. That also led to mutual confusion between the *Panus* and *Lentinus* species. With the application of phylogenetic analysis in researching these groups, the view held by Corner (1981) was confirmed; furthermore, this group was divided into three separate genera, viz. *Lentinus*, *Panus* and *Neolentinus* Redhead & Ginns (Hibbett and Vilgalys 1991; Thorn et al. 2000). Meanwhile, the family status of *Panus* has been changed several times and it has been placed in Agaricaceae, Polyporaceae, Tricholomataceae and Meruliaceae (Fries 1838; Imai 1938; Singer 1961a, 1961b, 1962; Miller 1972; Douanla-Meli and Langer 2010; Zmitrovich and Kovalenko 2016). However, in recent years, the taxonomic status of *Panus* has been significantly changed again, due to phylogenetic studies and it has been upgraded to a separate family rank, Panaceae (Justo et al. 2017).

Now, genus *Panus* could be clearly distinguished from the genus *Lentinus* because of its skeletal hyphae (Corner 1981; Pegler 1983). However, *Panus* is not easy to distinguish from the genus *Pleurotus* (Fr.) P. Kumm. It is well known that the fungi of the genus *Pleurotus* have unique nematode-feeding properties (Li and Bau 2014). Its softer texture, on the other hand, was once thought to be a monomitic hyphal system with only generative hyphae. In contrast, the fungi of *Panus* have a firmer texture with a typical dimitic hyphal system, which can be distinguished from *Pleurotus*. However, as research continued, many species in the genus *Panus* were found to have nematode-predatory functions, which corresponded precisely to the characteristics of *Pleurotus* and, thus, a large number of species were moved into *Pleurotus*, such as *Pleurotusgiganteus* (Berk.) Karun. & K.D. Hyde and *Pleurotustuber-regium* (Fr.) Singer (Singer 1951; Klomklung et al. 2012). Yet these species that have migrated into *Pleurotus* have the same dimitic hyphal system as *Panus*, which is quite different from the commonly known characteristics of *Pleurotus*. This has led to a blurring of the line between *Panus* and *Pleurotus*, making it difficult to distinguish.

In addition, there are some species similar in appearance, for example, *Lentinusciliatus* Lév., *Lentinussimilis* Berk. & Broome and *Lentinushookerianus* Berk. etc. These belonged to the sect. Velutini sensu Pegler and shared a similar appearance, because of its velutinate to strigose basidiomes and thick-walled skeletocystidia (Pegler 1983). However, later, four of them were recombined into *Panus*, due to the presence of skeletal hyphae and phylogenetic studies, leaving *L.velutinus* and *L.fasciatus* behind (Pegler 1983; May and Wood 1995), but these two species also show close affinity with genus *Panus* (Pegler 1983; Douanla-Meli and Langer 2010; Luangharn et al. 2019).

Due to the omission of the taxonomic studies, the species diversity of *Panus* is still unclear in China and lacks systematic research. Teng (1963) described three *Panus* species, viz. *Panustorulosus* (Pers.) Fr. (≡*P.conchatus*), *Panusrudis* Fr. and *Panussetiger* (Lév.) Teng in his book—*Fungi of China*. Later, Tai (1979) recorded four species, *P.conchatus*, *P.rudis*, *P.setiger* and *Panustigrinus* (Bull.) Singer (≡*Lentinustigrinus* (Bull.) Fr.), in “*Sylloge Fungorum Sinicorum*”. Subsequently, the species diversity of *Panus* has been performed locally in China (Li et al. 1978; Du et al. 1983; Xu et al. 1986; Li and Qin 1991; Li et al. 2004; Shao and Xiang 2006). In 2014, the first monograph about pleurotoid and lentinoid fungi in China was published, in which 11 species of *Panus* were recorded (Li and Bau 2014). Then, “*the Atlas of Chinese Macrofungal Resources*” described six species (Li et al. 2015).

Therefore, up to now, there are only a total of eight *Panus* species that have been recorded from China, viz. *Panusciliatus* (Lév.) T.W. May & A.E. Wood, *P.conchatus*, *Panusneostrigosus* Drechsler-Santos & Wartchow, *P.rudis*, *P.setiger*, *Panussimilis* (Berk. & Broome) T.W. May & A.E. Wood, *Panusstrigellus* (Berk.) Chardón & Toro and *Panusvelutinus* (Fr.) Sacc. (≡*L.velutinus*). In addition, most of them are located in southwest China, northwest China, south China, central China and Northeast China. *Panus* resources in east China and north China are in urgent need of development, especially in previously undocumented areas such as Zhejiang Province.

In this study, a combined morphological and phylogenetic study of six *Panus* species from China is carried out. Two new species, *Panusminisporus* and *Panusbaishanzuensis* and a new record, *P.similis*, from Zhejiang Province are described in detail, along with illustrations and colour photographs.

## ﻿Materials and methods

### ﻿Sampling and morphological studies

All examined specimens in this study were collected from China. The pictures of fruit-body were taken in the field. After measuring and describing the fresh macroscopic characteristics, the specimens were dried at 40–50 °C in a dryer, then they were stored in the Fungarium of Jilin Agricultural University (FJAU).

Macroscopic characteristics were based on the field notes and the colours were described according to Küppers (2002). The descriptions of size were referred to Yue et al. (2023). Then microscopic characteristics were observed from the dried specimens using a Zeiss Axio lab. A1 light microscope. The dried specimens were rehydrated in 94% ethanol first, then mounted in 3% potassium hydroxide (KOH) to observe the colour, sealed in 1% Congo red to measure the data and Melzer’s reagent was used to check if the spores were amyloid or dextrinoid (Hu et al. 2022a, b). As to each specimen, at least 40 values were measured separately from different basidiomata for each feature. The measurements are given as (a)b–c(d), the range of b–c contains a minimum of 90% of the measured values and the extreme values (i.e. a and d) are given in parentheses. The extent for basidiospores is given as length × width (L × W), Q values equal to the ratio of length and width of each basidiospore in the side view, “n” represents the number of measured basidiospores, “lm” represents the arithmetic mean of the length, “wm” represents the arithmetic mean of the width and “q” represents the average Q value of all basidiospores ± standard deviation.

### ﻿DNA extraction, PCR amplification and sequencing

The total DNA was extracted using the new plant genomic DNA extraction kit from Jiangsu Kangwei Century Biotechnology Company Limited, following the instructions. Primer pairs ITS1-F/ITS4-B (Gardes and Bruns 1993), LR0R/LR5 (Cubeta et al. 1991) and 983F/2212R (Rehner and Buckley 2005) were used for amplifying and sequencing these sequences: internal transcribed spacer (ITS) regions, large subunit nuclear ribosomal RNA gene (nLSU) and translation elongation factor 1-α gene (*tef-1α*), respectively. The amplification reactions were carried out in a total 25 μl system, which is as follows: dd H_2_O 13.5 μl, 2 × Es Taq MasterMix (Dye) 8 μl, 10 mM of each primer 1 μl and DNA solution 1.5 μl. The PCR reaction procedures were as follows: for ITS, 1) 95 °C for 2 min to initial denaturation, 2) 35 cycles of denaturation for 40 s at 94 °C, annealing for 1 min at 50 °C and extension for 2 min at 75 °C, 3) leave at 75 °C for 10 min (Zmitrovich and Kovalenko 2016); for nLSU, 1) 95 °C for 3 min to initial denaturation, 2) 30 cycles of denaturation for 30 s at 94 °C, annealing for 45 s at 47 °C and extension for 1 min and 30 s at 72 °C, 3) leave at 72 °C for 10 min (Hu et al. 2022b); and for *tef-1α*, 1) 95 °C for 2 min to initial denaturation, 2) 9 cycles of denaturation for 40 s at 95 °C, annealing for 40 s at 60 °C and extension for 2 min at 70 °C, 3) then 36 cycles of denaturation for 45 s at 95 °C, annealing for 1 min and 30 s at 50 °C and extension for 2 min at 70 °C, 4) leave at 70 °C for 10 min (Zmitrovich and Kovalenko 2016). The PCR products were detected by 1.2% agarose gel electrophoresis, then Comate Bioscience (Jilin) Company Limited was employed to carry out purification and sequencing. Finally, the sequencing results were uploaded to GenBank (https://www.ncbi.nlm.nih.gov/genbank/), Table 1.

### ﻿Data analysis

By searching in GenBank, 68 ITS sequences, 46 nLSU sequences and 23 *tef-1α* sequences of related taxa were downloaded. A total of 10 ITS sequences, 10 nLSU sequences and three *tef-1α* sequences were newly obtained in this study. All sequences used in this paper are listed in Table 1. Each single-gene dataset was aligned in MAFFT 7 using the E-INS-i strategy (Katoh and Standley 2013) and manually adjusted where necessary in BioEdit 7.0.9 (Hall 1999). The datasets (ITS+nLSU+*tef-1α*) were then concatenated using PhyloSuite 1.2.3 (Zhang et al. 2020; Xiang et al. 2023) for multi-phylogenetic analyses. ModelFinder 2.2.0 (Kalyaanamoorthy et al. 2017) was used to select the best-fit partition model (Edge-linked).

**Table 1.** Voucher/specimen ID, GenBank accession numbers and origin of the specimens included in this study. Sequences produced in this study are in bold.

Taxon	Voucher/ specimen ID	GenBank Accession Number			Origin	Reference
		ITS (5.8S)	nLSU	*tef-1α*		
*Agaricuscampestris*	MA Fungi 80998	NR_151745	-	-	USA	Unpublished
*A.campestris*	LAPAG370	KM657927	KR006607	KR006636	China	Zhou et al. (2016)
*Antellaamericana*	HHB-4100-Sp	KP135316	KP135196	-	USA	Floudas and Hibbett (2015)
*Antrodiellastipitata*	FD-136	KP135314	KP135197	-	USA	Floudas and Hibbett (2015)
*Aurantiporusalbidus*	CIEFAP-117	KY948739	KY948848	-	USA	Justo et al. (2017)
*Boletusedulis*	HMJAU4637	-	KF112455	KF112202	China	Wu et al. (2014)
*Coniophoraarida*	FP-104367	GU187510	GU187573	GU187684	USA	Binder et al. (2010)
*Fomitiporellaaustroasiana*	Dai 16244	MG657328	MG657320	-	China	Ji et al. (2018)
*Fulvifomeshainanensis*	Dai 11573	KC879263	JX866779	-	China	Zhou (2014)
*Gloeophyllumsepiarium*	Wilcox-3BB	HM536091	HM536061	HM536110	USA	Garcia-Sandoval et al. (2011)
*G.trabeum*	1320	HM536094	HM536067	HM536113	USA	Garcia-Sandoval et al. (2011)
*Hydnoporiasubrigidula*	He1157	JQ716403	JQ716409	-	China	He and Li (2013)
*Hygrophoropsisaurantiaca*	AFTOL-ID 714	AY854067	AY684156	AY883427	USA	Matheny et al. (2006)
*Hymenochaetebambusicola*	He 4116	KY425674	KY425681	-	China	Nie et al. (2017)
*Hyphodermellarosae*	FP-150552	KP134978	KP135223	-	USA	Floudas and Hibbett (2015)
*Imleriabadius*	MB 03-098a	-	KF030355	KF030423	USA	Nuhn et al. (2013)
*Irpexlacteus*	FD-9	KP135026	KP135224	-	USA	Floudas and Hibbett (2015)
*Lentinulaboryana*	TENN58368	MW508930	-	MW553225	Brazi	Menolli et al. (2022)
*L.edodes*	TMI1633	MW508938	-	MW553232	Thailand	Menolli et al. (2022)
*L.madagasikarensis*	PC0142531	MW810301	MW810299	OK598120	Madagascar	Menolli et al. (2022)
*L.raphanica*	TENN56555	MW508963	-	MW553250	Costa Rica	Menolli et al. (2022)
*Lentinuscrinitus*	SA37	OK393677	OK383448	-	Brazil	Unpublished
*L.tigrinus*	LE214778	KM411459	KM411475	KM411490	Russia	Zmitrovich and Kovalenko (2016)
*Meruliushydnoidea*	HHB-1993-sp	KY948778	KY948853	-	USA	Justo et al. (2017)
*Neolentinusadhaerens*	DAOM 214911	HM536096	HM536071	HM536117	Canada	Garcia-Sandoval et al. (2011)
*Neolentinuskauffmanii*	DAOM 214904	HM536097	HM536073	HM536118	USA	Garcia-Sandoval et al. (2011)
*Oxyporuscorticola*	ZRL20151459	LT716075	KY418899	-	China	Zhao et al. (2017)
***Panusbaishanzuensis***	**FJAU67793**	**PP273985**	**PP273975**	**PP590553**	**China**	**This study**
***P.baishanzuensis***	**FJAU67793**	**PP273986**	**PP273976**	**PP590554**	**China**	**This study**
***P.baishanzuensis***	**FJAU67793**	**PP273987**	**PP273977**	**PP590555**	**China**	**This study**
*P.ciliatus*	SP446150	MT669118	MT669140	-	Brazil	Unpublished
***P.conchatus***	**FJAU67795**	**PP273979**	**PP273969**	**PP590545**	**China**	**This study**
*P.conchatus*	LE265028	KM411463	KM434323	KM411496	Russia	Zmitrovich and Kovalenko (2016)
*P.fulvus*	DS1687	MT669122	MT669143	-	Brazil	Unpublished
***P.minisporus***	**FJAU67792**	**PP273980**	**PP273970**	**PP590550**	**China**	**This study**
***P.minisporus***	**FJAU67792**	**PP273981**	**PP273971**	**PP590551**	**China**	**This study**
***P.minisporus***	**FJAU67792**	**PP273982**	**PP273972**	**PP590552**	**China**	**This study**
***P.neostrigosus***	**FJAU67796**	**PP273983**	**PP273973**	**PP590547**	**China**	**This study**
*P.neostrigosus*	LE5829	KM411451	KM411468	KM411483	Russia	Zmitrovich and Kovalenko (2016)
*P.parvus*	URM80840	MT669125	MT669145	-	Brazil	Unpublished
***P.rudis***	**FJAU7824**	**PP273988**	**PP273978**	**PP590546**	**China**	**This study**
*P.rudis*	ZJ1005DKJO2	KU863049	OR772972	-	China	Luangharn et al. (2019)
***P.similis***	**FJAU67794**	**PP273984**	**PP273974**	**PP590548**	**China**	**This study**
*P.similis*	LE287548	KM411466	KM411482	-	Russia	Zmitrovich and Kovalenko (2016)
*P.strigellus*	INPA239979	JQ955724	JQ955731	-	Brazil	Vargas-Isla et al. (2015)
*P.tephroleucus*	CMINPA 1860	MN602052	-	-	Brazil	Unpublished
*P.velutinus*	VOG30	MT669139	MT669155	-	Brazil	Unpublished
*Phanerochaeteaustralis*	HHB-7105-Sp	KP135081	KP135240	-	USA	Floudas and Hibbett (2015)
*Pleurotusabieticola*	6554	AY450348	-	-	Russia	Petersen and Hughes (1997)
*Pl.australis*	VT1953	AY315758	-	-	Australia	Zervakis et al. (2004)
*Pl.calyptratus*	HMAS 63355	AY562495	AY562496	-	China	Luangharn et al. (2019)
*Pl.citrinopileatus*	FSCC1 (PCY1)	JN234853	-	-	Malaysia	Avin et al. (2012)
*Pl.cornucopiae*	H-14	JQ837484	-	-	Russia	Shnyreva et al. (2012)
*Pl.cystidiosus*	D419	AY315774	-	-	USA	Zervakis et al. (2004)
*Pl.djamor*	CBS 665.85	EU424288	EU365645	-	China	Luangharn et al. (2019)
*Pl.dryinus*	ECS-1108	GU722278	-	-	Mexico	Huerta et al. (2010)
*Pl.eous*	P109	MG282448	-	-	South Korea	Unpublished
*Pl.eryngii*	LGAMP63	HM998811	-	-	Greece	Zervakis et al. (2014)
*Pl.euosmus*	CBS 307.29	EU424298	EU365654	-	United Kingdom	Unpublished
*Pl.fossulatus*	D1821	EU233946	U04136	-	USA	Vilgalys and Sun (1994)
*Pl.fuscosquamulosus*	LGAMP50	AY315789	-	-	Greece	Zervakis et al. (2004)
*Pl.giganteus*	CMU54-1	JQ724360	JQ724361	-	Thailand	Kumla et al. (2013)
*Pl.nebrodensis*	UPA28	HM998818	-	-	Italy	Zervakis et al. (2014)
*Pl.opuntiae*	SAF 251	MH620771	MK182780	-	Italy	Zervakis et al. (2019)
*Pl.ostreatus*	TENN 53662	AY854077	AY645052	AY883432	USA	Unpublished
*Pl.placentodes*	HKAS57781	KR827694	KR827696	KR827700	China	Liu et al. (2016)
*Pl.populinus*	ATCC 90083	AY368667	-	-	USA	Zervakis et al. (2019)
*Pl.pulmonarius*	ICMP 18163	MH395973	MH395998	-	New Zealand	Unpublished
*Pl.tuber-regium*	FRI 3611	KX018290	-	-	Malaysia	Karunarathna et al. (2016)
*Polyporustuberaster*	MUCL31757	-	AB368103	-	Japan	Sotome et al. (2008)
*Pterulaecho*	DJM302S58	DQ494693	AY629315	GU187743	USA	Matheny et al. (2006)
*Radlodonyunnanensis*	BJFC 010487	NR_182985	-	OM982705	China	Wang and Dai (2022)
*R.americanus*	RLG6350	JQ070175	-	-	USA	Nakasone and Lindner (2012)
*R.casearius*	CLZhao 3796	MH114880	-	-	China	Unpublished
*Serpulalacrymans*	REG_383	GU187542	GU187596	GU187752	USA	Binder et al. (2010)
*Steccherinumbourdotii*	HHB-9743-sp	KY948818	-	-	USA	Justo et al. (2017)
*Trametopsiscervina*	AJ-185	JN165020	JN164796	-	USA	Justo and Hibbett (2011)
*Tricholomaflavovirens*	AP2l	EU186294	-	EU186270	Portugal	Unpublished
*T.megalophgeum*	WTU F:073091	NR_175704	-	-	USA	Unpublished
*Veluticepsafricana*	CBS 403.83	MH861619	-	-	Gabon	Vu et al. (2019)
*V.berkeleyi*	HHB-8594-Sp	HM536099	HM536081	HM536126	USA	Garcia-Sandoval et al. (2011)

The datasets were analysed separately using Maximum Likelihood (ML) and Bayesian Inference (BI). For the ML analysis, it was performed using IQ-Tree 1.6.12 (Schmidt et al. 2014). The tree topology was verified under both 1000 bootstrap and 1000 replicates of the SH-aLRT branch test. For the BI analysis, it was performed using MrBayes 3.2.6 (Ronquist et al. 2012). The analysis employed a general time-reversible DNA substitution model and a gamma distribution to account for rate variation across the sites. Four Markov chains were executed for two runs, starting from random trees, the chains being terminated when the split deviation frequency value fell below 0.01. Tree samples were sampled every 1000 generations. The first 25% of the sampled trees were discarded as burn-in, while the remaining trees were used to construct a 50% majority consensus tree and to calculate the Bayesian posterior probabilities (BIPP). Then the phylogenetic trees were visualised using FigTree 1.4.3 (Andrew 2016).

## ﻿Results

### ﻿Phylogenetic analyses

**Figure 1.**
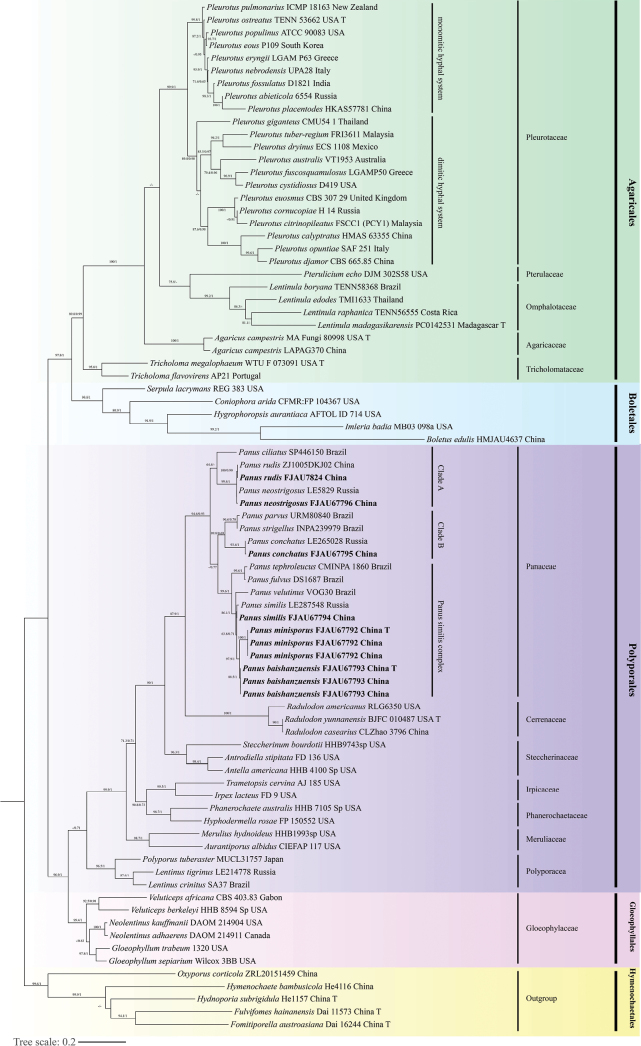
The 50% majority rule Maximum Likelihood analysis of *Panus* and the related groups, based on ITS, nLSU and *tef-1α* sequences, with Hymenochaetales as outgroup. Support values of internal nodes respectively represent the Maximum Likelihood bootstrap (MLBP ≥ 70) and Bayesian posterior probability (BIPP ≥ 70%). The Voucher or specimen ID and the country are marked after the species name and the sequence from the type specimen is also marked as “T” at the end.

Three gene fragments (ITS, nLSU and *tef-1α*) from representative species of five orders in the Agaricomycetes were selected to construct a phylogenetic analysis, which included 71 species belonging to 31 genera of 19 families. The aims are to explore the phylogenetic status within *Panus* and its relationship with allies. A total of 160 sequences were used for phylogenetic analysis in this study. The best-fit model is GTR+F+I+G4, TIM3+F+I+G4, TPM3+TNe+I for ITS, nLSU and *tef-1α* datasets, respectively. The topologies of ML and BI, based on the concatenated datasets, were consistent and typically increased support values; thus, only the result inferred from ML analysis was displayed (Fig. 1).

In the phylogenetic tree, four clades corresponding to Agaricales, Boletales, Polyporales and Gloeophyllales were revealed, with Hymenochaetales as outgroup (Fig. 1). As indicated in the tree, four families were selected as representatives of Agaricales. It is worth noting that *Pleurotus* is divided into two deeply-divergent clades. One represents the monomitic hyphal system species and the other is the dimitic hyphal system species (Fig. 1). Boletales is located between Agaricales and Polyporales. In the Polyporales clade, a total of seven families were selected to reconstruct the phylogenetic analysis, with the family Panaceae as the main group (Fig. 1). Amongst them, the genus *Panus* is divided into three clades, viz. clade A, clade B and *P.similis* complex clade. Thirty-one specimens we sampled formed two new species, *Panusminisporus* L. Yue, J.J. Hu, B. Zhang & Y. Li, sp. nov. and *Panusbaishanzuensis* L. Yue, B. Zhang & Y. Li, sp. nov., one new record species from Zhejiang Province, *P.similis* and three common species, *P.conchatus*, *P.neostrigosus* and *P.rudis*, which were clustered in *Panus*. Further morphological research of other related species was consistent with supporting the classification of these two new species. Additionally, the order Gloeophyllales is located between the orders Polyporales and Hymenochaetales (Fig. 1).

### ﻿Taxonomy

#### ﻿ Panus minisporus

Taxon classificationFungiPolyporalesPanaceae

L. Yue, J.J. Hu, B. Zhang & Y. Li sp. nov.

45244E56-4B89-501D-A33B-111D391F48AA

Fungal Names: FN 571875

Figs 2 , 3

##### Etymology.

The epithet ‘minisporus’ refers to the small basidiospores of the new species.

##### Diagnosis.

This species is distinguished from closed species by the cyathiform or flared and reddish-brown (N_60_Y_90_M_60_) pileus, white or dirty white (N_00_Y_10-20_M_00-10_) lamellae with cross-veins and two tiers of lamellulae, slender stipe, smaller basidiospores, wider generative hyphae and absence of sclerocystidia.

##### Holotype.

China. Guizhou Province: Qiannan Buyi and Miao Autonomous Prefecture, Libo County, Maolan National Nature Reserve, 25.32°N, 108.08°E, 8 August 2017, Jiajun Hu, FJAU67792 (GenBank accession no.: ITS: PP273980, PP273981, PP273982; nLSU: PP273970, PP273971, PP273972; *tef-1α*: PP590550, PP590551, PP590552).

##### Description.

Basidiomata solitary, large. Pileus 2.5–6.5 cm in diameter, thin, coriaceous, applanate, cyathiform or flared, reddish-brown (N_60_Y_90_M_60_), darker at the centre, covered with reddish-brown (N_60_Y_90_M_60_) puberulent, stripe dense and slender, radially parallel distributed, margin integer and ciliate slightly dense. Lamellae decurrent, crowded, white or dirty white (N_00_Y_10-20_M_00-10_), with cross-veins and two tiers of lamellulae, edge entire. Stipe 4.4–9 × 0.2–0.5 cm, inverted clavate, central, solid, coriaceous, surface reddish-brown (N_60_Y_90_M_60_) or more often darker, with dense velutinus, slightly expanded at the base. Pseudosclerotium absent. Context thin, up to 1 mm thick, white (N_00_Y_10_M_00_), coriaceous, consisting of a dimitic hyphal system with skeletal hyphae.

**Figure 2.**
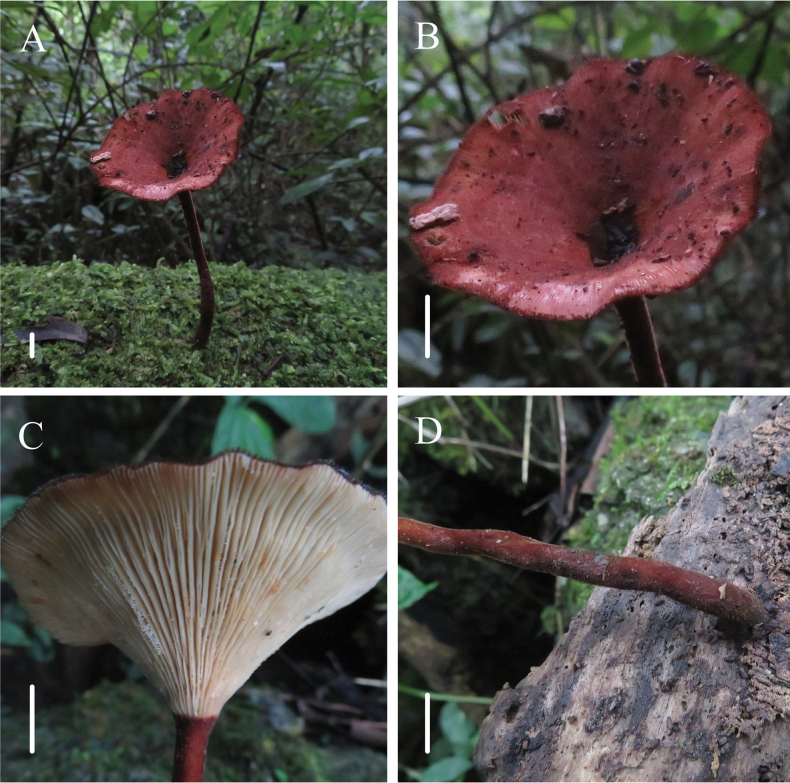
Habitat of *Panusminisporus* (FJAU67792, holotype) **A** basidiocarp **B** pileus **C** lamellae **D** stipe. Scale bars: 1 cm.

Generative hyphae 3–5(7) μm diameter, cylindrical, not inflated, hyaline, thin-walled, frequently branched, with prominent clamp connections. Skeletal hyphae 2–3 μm diameter, sinuous cylindrical, hyaline, with a thick-walled and continuous lumen, unbranched. Basidiospores 4.5–5(5.5) × 2.5-3 μm (n = 40, lm = 5 μm, wm = 3 μm, Q = 1.5–1.83, q = 1.67), ellipsoid to oblong, smooth, hyaline, thin-walled. Basidia (16)19–26 × 5–6 μm, clavate or elongated, bearing 4 sterigmata. Lamella-edge sterile, with small cheilocystidia. Cheilocystidia crowded, 16–22 × 5–6 μm, with median constriction, nodulose-clavate, fusoid, irregular, hyaline, thin-walled. Sclerocystidia absent. Hymenophoral trama irregular, radiate construction, hyaline, similar in structure to the context. Pileipellis on epicutis, made up of thick-walled generative hyphae, 3–5.5(7) μm diameter, occasionally bunched, not inflated, light brown. Stipitipellis similar to pileipellis.

**Figure 3.**
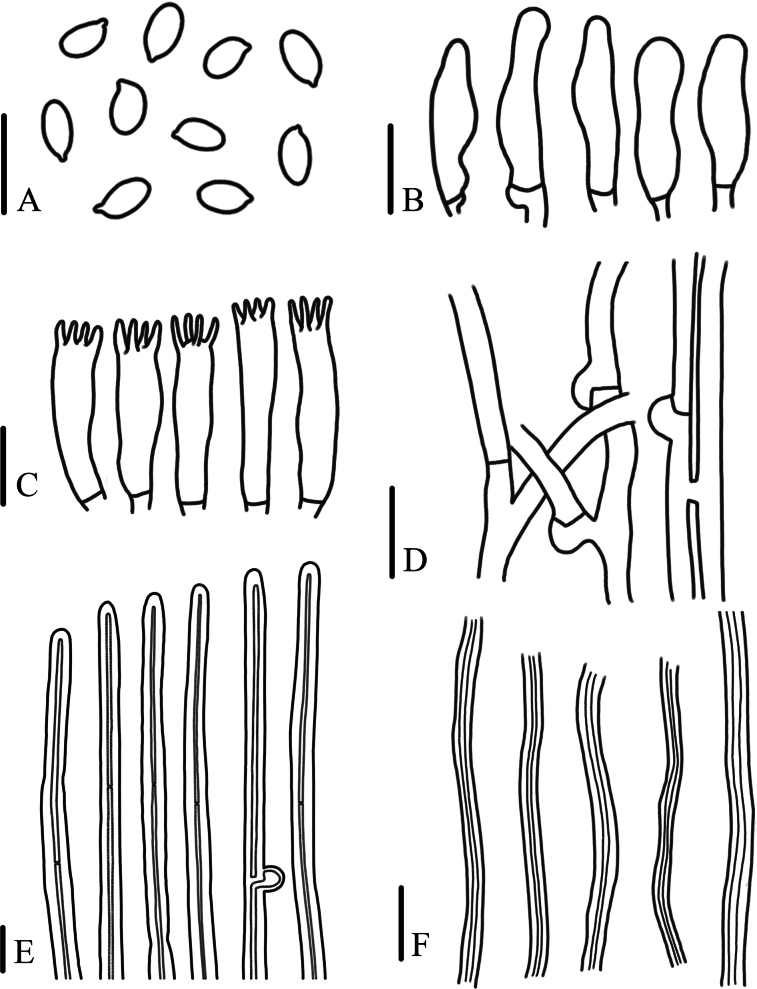
Microscopic characteristics of *Panusminisporus* (FJAU67792, holotype) **A** basidiospores **B** cheilocystidia **C** basidia **D** generative hyphae of context **E** pileipellis hyphae **F** skeletal hyphae of context. Scale bars: 10 μm.

##### Ecology.

Solitary on rotten wood in broad-leaved forest.

##### Distribution.

China (Guizhou Province).

##### Notes.

This species is characterised by the reddish-brown pileus and stipe, white or dirty white lamellae with cross-veins and two tiers of lamellulae, slender stipe, smaller basidiospores and absence of sclerocystidia.

*Panusminisporus* is close to *P.velutinus* and *P.similis* in morphology, because of the velutinate pileus and slender stipe. However, the pileus and stipe of *P.minisporus* are both reddish-brown, which is different from the pale greyish-cinnamon to rufous or tawny-brown tints of *P.velutinus* and the cinnamon-brown to dark chestnut-brown, with violaceous or purplish tints of *P.similis*. Meanwhile, the lamellae of *P.minisporus* are white or dirty white, with cross-veins and two tiers of lamellulae, but the lamellae of *P.velutinus* and *P.similis* have no cross-veins and with 3 or 4 and 5 tiers of lamellulae, respectively. In addition, the pseudosclerotium of *P.minisporus* is absent, but *P.velutinus* and *P.similis* often have distinct pseudosclerotium. In terms of micromorphology, *P.minisporus* has smaller spores and Q values, wider generative hyphae and absent sclerocystidia, all of which can be distinguished from *P.velutinus* and *P.similis*.

#### ﻿ Panus baishanzuensis

Taxon classificationFungiPolyporalesPanaceae

L. Yue, B. Zhang & Y. Li sp. nov.

82F40A3A-3129-507D-9BFF-E9E3CB875B5D

Fungal Names: FN 571876

Figs 4 , 5

##### Etymology.

The epithet ‘baishanzuensis’ refers to the type locality, Baishanzu National Park, of this species.

##### Diagnosis.

This species differs from closely-related species by pileus with concentric darker zones, crowded lamellae with cross-veins, shorter stipe, lack of pseudosclerotium, longer basidiospores, greater Q values, diverse and shorter cheilocystidia and smaller sclerocystidia.

##### Holotype.

China. Zhejiang Province: Lishui City, Qingyuan County, Baishanzu National Park, 27.62°N, 118.92°E, 28 July 2023, Yingkun Yang & Lei Yue, FJAU67794 (GenBank accession no.: ITS: PP273985, PP273986, PP273987; nLSU: PP273975, PP273976, PP273977; *tef-1α*: PP590553, PP590554, PP590555).

##### Description.

Basidiomata solitary, medium. Pileus 6–6.8 cm in diameter, thin, coriaceous, infundibuliformis or flared, ochre-brown (N_70_Y_99_M_60_), with concentric darker zones, densely covered with brown (N_50_Y_80_M_30_) farinaceus pilosus, stripe dense and slender, radially parallel distributed, margin sinuatus. Lamellae decurrent, crowded, pale yellow (A_60_M_00_C_00_), with cross-veins and six tiers of lamellulae, edge entire. Stipe 2.3–3.1 × 0.3–0.6 cm, short clavate, excentric, solid, coriaceous, surface concolorous with the pileus or more often darker, with densely velutinus, slightly expanded at the apex. Pseudosclerotium absent. Context thin, up to 1 mm thick, white (N_00_Y_10_M_00_), coriaceous, consisting of a dimitic hyphal system with skeletal hyphae.

**Figure 4.**
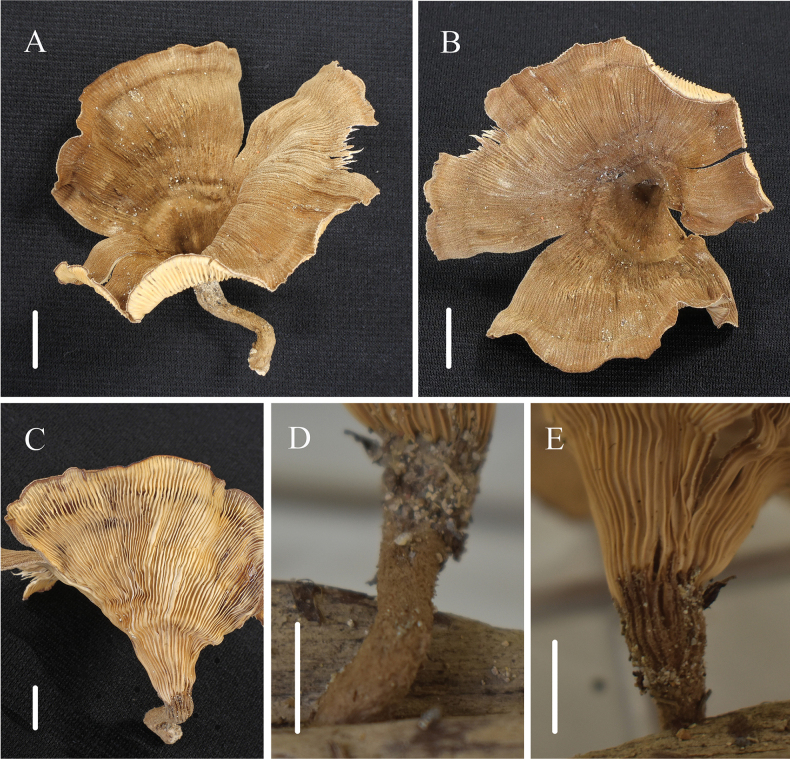
Habitat of *Panusbaishanzuensis* (FJAU67794, holotype) **A** basidiocarp **B** pileus**C** lamellae **D, E** stipe. Scale bars: 1 cm.

Generative hyphae 2–3 μm diameter, cylindrical, not inflated, hyaline, thin-walled, frequently branched, with prominent clamp connections. Skeletal hyphae 2–3 μm diameter, sinuous cylindrical, hyaline, with a thick-walled and continuous lumen, unbranched. Basidiospores 7–8(9) × 3–3.5 μm (n = 40, lm = 7.6 μm, wm = 3.07 μm, Q = 2.14–2.67, q = 2.48), cylindrical, smooth, hyaline, thin-walled. Basidia (16)20–25 × 5.5–7 μm, clavate or elongated, bearing four sterigmata. Lamella-edge sterile, with short cheilocystidia. Cheilocystidia crowded, (12)14–19 × (5.5)6–7(8) μm, with median constriction, ellipsoid or utriform and apical protrusion, hyaline, thin-walled. Sclerocystidia abundant, (16.5)19–24 × (5)5.5–6.5(7) μm, clavate to irregularly fusoid, with a thickened wall, hyaline. Hymenophoral trama irregular, radiate construction, hyaline, similar with context. Pileipellis epicutis, made up of thick-walled generative hyphae, 3–5 μm diameter, occasionally brunched, not inflated, light brown. Stipitipellis similar to pileipellis.

**Figure 5.**
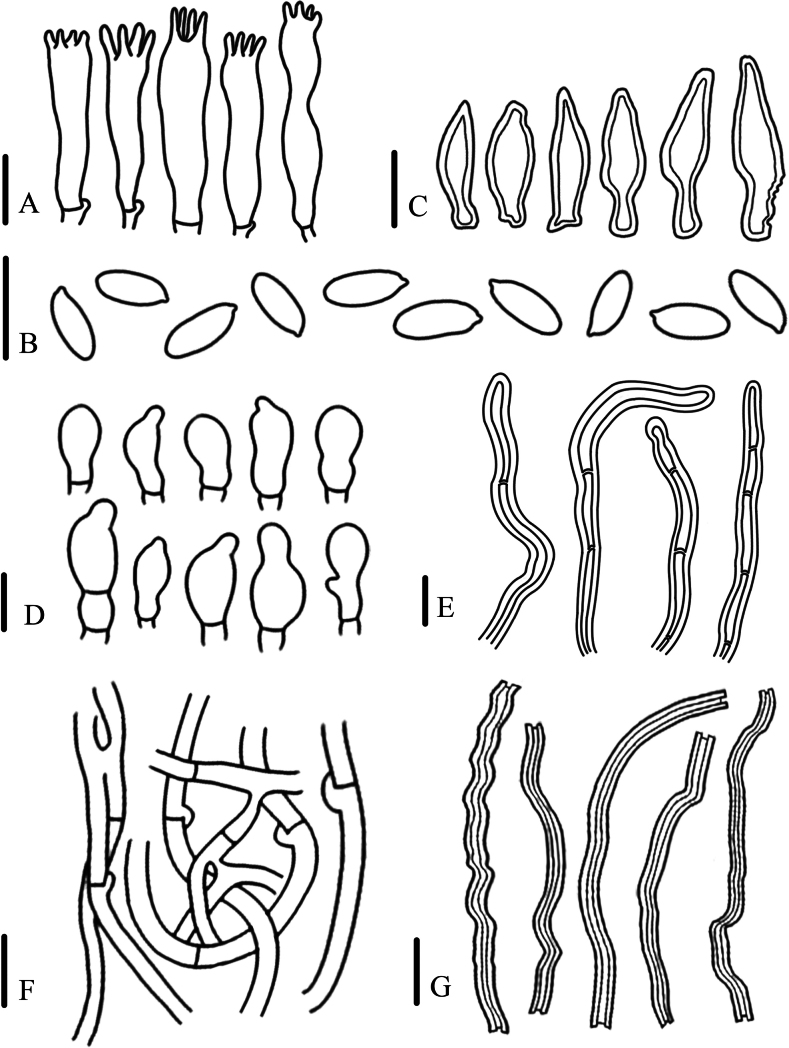
Microscopic characteristics of *Panusbaishanzuensis* (FJAU67794, holotype) **A** basidia **B** basidiospores **C** sclerocystidia **D** cheilocystidia **E** pileipellis hyphae **F** generative hyphae of context **G** skeletal hyphae of context. Scale bars: 10 μm.

##### Ecology.

Solitary on rotten wood in broad-leaved forest.

##### Distribution.

China (Zhejiang Province).

##### Notes.

This species is characterised by the concentric darker zones pileus, decurrent lamellae with cross-veins, shorter stipe, longer basidiospores, diverse and shorter cheilocystidia and smaller sclerocystidia.

*Panusbaishanzuensis* is close to *P.similis* and *P.velutinus* in morphology, because of the similar pileus and lamellae. However, the pileus of *P.velutinus and P.similis* without concentric darker zones distinguished them from *P.baishanzuensis*. Meanwhile, the stipe of *P.baishanzuensis* is extremely short, while the stipe of *P.velutinus* and *P.similis* is long and slender. In addition, the lamellae of *P.baishanzuensis* have cross-veins with six tiers of lamellulae, but *P.velutinus* and *P.similis* have no cross-veins and with three or four and five tiers of lamellulae. At the same time, *P.velutinus* and *P.similis* both have pseudosclerotia, while the pseudosclerotium of *P.baishanzuensis* is absent. Lastly and most importantly, *P.baishanzuensis* has longer basidiospores, graeter Q values, shorter cheilocystidia and smaller sclerocystidia than *P.similis* and *P.velutinus*.

### ﻿New record of Zhejiang Province, China

#### ﻿ Panus similis

Taxon classificationFungiPolyporalesPanaceae

(Berk. & Broome) T.W. May & A.E. Wood, Mycotaxon 54: 148 (1995)

852E6E08-E428-59FF-9806-2A0720E2DDF3

Figs 6 , 7

##### Description.

Basidiomata solitary, medium to large. Pileus 3–5.5 cm in diameter, thin, coriaceous, infundibuliform to cyathiform, cinnamon-brown or pale brown (N_50_Y_40-60_M_20-40_), glabrous, radially plicate-sulcate with striae extending almost to the centre, without concentric zones; margin curved, ciliate not apparent. Lamellae decurrent, crowded, neither furcate nor anatomosing, buff or pale brown (N_10_A_50-60_M_10-20_), with five tiers of lamellulae, edge entire. Stipe 7.5–9 × 0.35–0.9 cm, clavate, central, solid, coriaceous, surface chestnut brown, with velutinus, slightly expanded at base. Pseudosclerotium slightly small, irregular. Context thin, up to 1 mm thick, white (N_00_Y_10_M_00_), coriaceous, consisting of a dimitic hyphal system with skeletal hyphae.

Generative hyphae 3–5.5 μm wide, cylindrical, not inflated, hyaline, thin-walled, frequently branched, with prominent clamp connections. Skeletal hyphae 2–3 μm diameter, sinuous cylindrical, with hyaline or pale brown thick-walled and continuous lumen, unbranched. Basidiospores 5.5–7 × 3–3.5 μm (n = 40, lm = 6.24 μm, wm = 3.03 μm, Q = 1.57–2.33, q = 2.06), cylindrical, smooth, hyaline, thin-walled. Basidia (18)20–25 × (4)5–6 μm, clavate, cylindrical, bearing four sterigmata. Lamella-edge sterile, with smaller cheilocystidia. Cheilocystidia crowded, (13)14–21 × 5.5–7 μm, nodulose-clavate, irregular, hyaline, thin-walled. Sclerocystidia abundant, 21(22)–32(35) × 5–6(6.5) μm, clavate to irregularly fusoid, with a thick, hyaline or brownish wall. Hymenophoral trama irregular, of radiate construction, hyaline, similar to context. Pileipellis epicutis, made up of thick-walled generative hyphae, 5–6.5 μm wide, occasionally bunched, not inflated, light brown. Stipitipellis similar to pileipellis.

**Figure 6.**
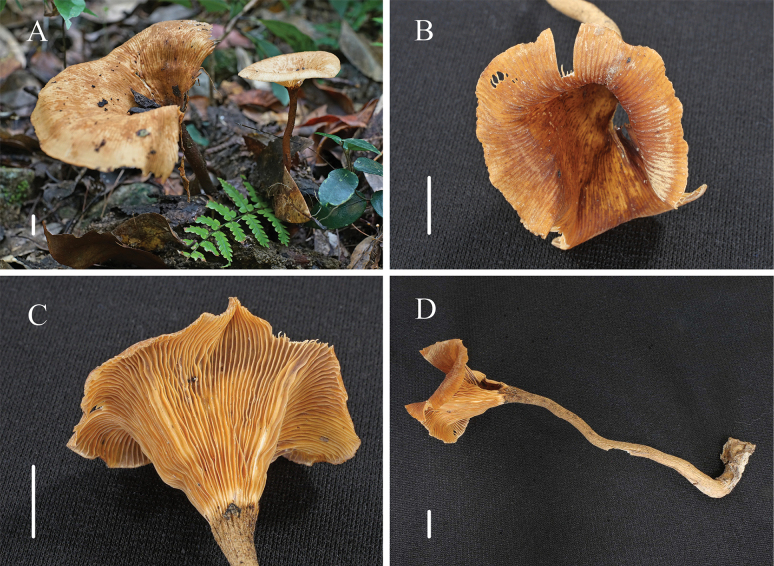
Habitat of *Panussimilis* (FJAU67793) **A** basidiocarps **B** pileus **C** lamellae **D** stipe. Scale bars: 1 cm.

##### Ecology.

Solitary on rotten wood in broad-leaved forest.

##### Distribution.

Angola, Australia, Brunei, China, Congo, India, Ivory Coast, Kenya, Malay Peninsula, Papua New Guinea, Philippines, Sabah, Sarawak, Sri Lanka, Tanzania, Thailand, Uganda, Vietnam, Zanzibar.

**Figure 7.**
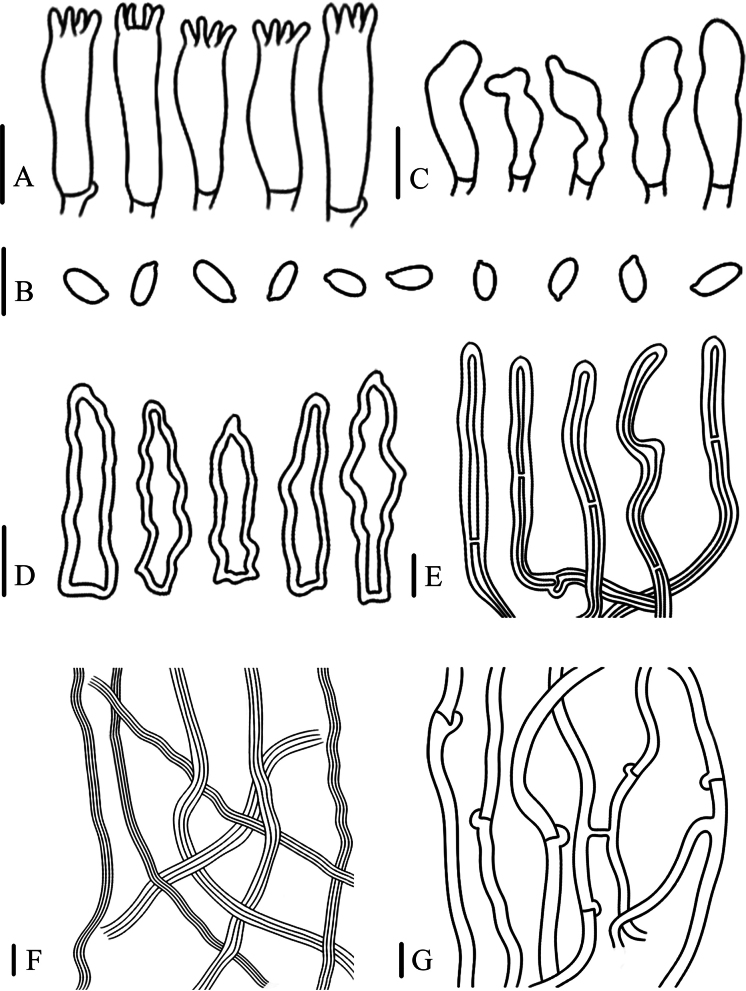
Microscopic characteristics of *Panussimilis* (FJAU67793) **A** basidia **B** basidiospores **C** cheilocystidia **D** sclerocystidia **E** pileipellis hyphae **F** skeletal hyphae of context **G** generative hyphae of context. Scale bars: 10 μm.

##### Specimen examined.

China. Zhejiang Province: Lishui City, Qingyuan County, Baishanzu National Park, 27.62°N, 118.92°E, 28 July 2023, Yingkun Yang & Lei Yue, FJAU67793.

##### Notes.

This species was originally described by Berkeley and Broome (1873) as *Lentinussimilis* Berk. & Broome, then it was treated as Panusfulvusvar.similis (Berk. & Broome) Corner (Corner 1981). However, Pegler (1983) disagreed with Corner and still accepted it as a member of *Lentinus*. Until 1995, it was first raised to a species rank as *P.similis* (May and Wood 1995).

Based on morphological research, there are some differences between our collected specimen and the original description.

Our specimen has distinct plicate-sulcate similar to the original description; however, its pseudosclerotium is smaller, whereas the original is very large. In addition, its hyphae structure is the same as that of the original description, but its spores are larger and its cheilocystidia are smaller compared to the latter.

Before this study, this species was not recorded from Zhejiang Province, China; thus it is the first report of *P.similis* from Zhejiang Province.

#### ﻿ Panus conchatus

Taxon classificationFungiPolyporalesPanaceae

(Bull.) Fr., Epicr. Syst. mycol. (Upsaliae): 396 (1838) [1836–1838]

C39B33AC-A359-5FFE-8269-6A91F82B7C29

Fig. 8A, B

##### Ecology.

Solitary on rotten wood.

##### Distribution.

Austria, Belgium, Bulgaria, China, Denmark, Eire, England, Estonia, Germany, India, Norway, Philippines, Russia, Scotland, Sri Lanka, Sweden and Wales.

##### Specimens examined.

China. Jilin Province: Baishan City, Fusong County, Quanyang Town, 4 July 2019, Bo Zhang & Jiajun Hu, FJAU67795; Baishan City, Fusong County, Quanyang Town, 4 July 2019, Bo Zhang & Jiajun Hu, FJAU67797; Baishan City, Fusong County, Quanyang Town, 4 July 2019, Bo Zhang & Jiajun Hu, FJAU67798; Dunhua City, Hancongling Scenic Area, 5 July 2019, Bo Zhang & Jiajun Hu, FJAU67799; Sichuan Province: Ganzi Tibetan Autonomous Prefecture, Jiulong County, Baitai Mountain, 16 July 2023, Xiaolan He, SAAS4904.

##### Notes.

*Panus* is typified as this species and is widely distributed worldwide. It is recorded from Hainan, Hunan, Inner Mongolia etc. from China (Li and Bau 2014).

The appearance of this species is varied. The stipe is easily influenced by the environment, from short to long. Additionally, when aged, due to the appearance of skeletal hyphae, it becomes tough from the soft flesh.

**Figure 8.**
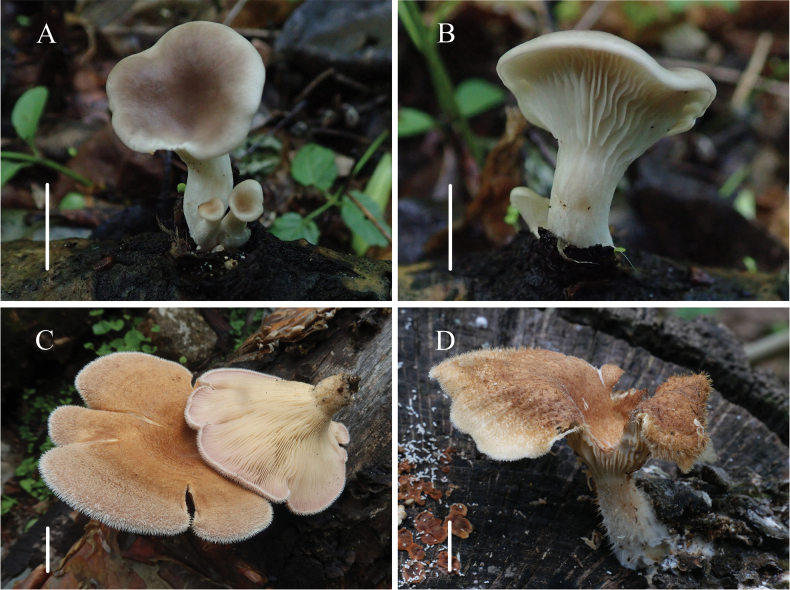
Habitat of *Panus* common species **A, B***Panusconchatus***C***Panusneostrigosus***D***Panusrudis*. Scale bars: 1 cm.

#### ﻿ Panus neostrigosus

Taxon classificationFungiPolyporalesPanaceae

Drechsler-Santos & Wartchow, J. Torrey bot. Soc. 139(4): 438 (2012)

7DBE188F-FC38-5E17-938F-AC26F82F1E5A

Fig. 8C

##### Ecology.

Solitary on rotten wood.

##### Distribution.

Argentina, Aru, Australia, Austria, Brazil, Bulgaria, Burma, Canada, China, Colombia, Cuba, Dominica, French Guiana, Germany, Galapagos, Hungary, Iran, India, Japan, Madagascar, Malay Peninsula, Mexico, Nepal, New Britain, Pakistan, Papua New Guinea, Philippine, Romania, Sabah, Santo Domingo, Sri Lanka, Russia, Thailand, Trinidad, Turkey, Uganda, U.S.A., Venezuela and Zaire.

##### Specimens examined.

China. Henan Province: Zhumadian City, Biyang County, Minzhuang Forest Farm, 32.52°N, 113.36°E, 2 August 2023, Yajie Liu, FJAU67796; Zhumadian City, Biyang County, Yihezhai Forest Farm, 32.39°N, 113.34°E, 19 August 2022, Yajie Liu, FJAU67800; Sichuan Province: Ganzi Tibetan Autonomous Prefecture, Jiulong County, Baitai Mountain, 16 July 2023, Xiaolan He, FJAU67801.

##### Notes.

This species is one of the most widely distributed in the genus *Panus*, which was originally described as *Lentinusstrigosus* Fr. (Fries 1825) in North Carolina, USA and was transferred to *Panus* by Drechsler-Santos et al. (2012). Due to its densely villous to hispid-strigose pileus, this species is often confused with *P.similis* complex. However, it can be distinguished from the complex by its metuloids.

#### ﻿ Panus rudis

Taxon classificationFungiPolyporalesPanaceae

Fr., Epicr. Syst. mycol. (Upsaliae): 398 (1838) [1836–1838]

95D719A8-E941-5871-9C45-CB4CA5FE00F4

Fig. 8D

##### Ecology.

Solitary on rotten wood.

##### Distribution.

Brazil, China, France and the Czech Republic (Czechia).

##### Specimens examined.

China. Inner Mongolia Autonomous Region: Xing’an League, Arshan City, Yiershi Town, 47.29°N, 119.84°E, 9 September 2002, Tolgor Bau, FJAU7824; Henan Province: Zhumadian City, Biyang County, Minzhuang Forest Farm, 32.52°N, 113.36°E, 2 August 2023, Yajie Liu, FJAU67802; Zhumadian City, Biyang County, Yihezhai Forest Farm, 32.39°N, 113.35°E, 31 August 2022, Yajie Liu, FJAU67803.

##### Notes.

This species is extremely similar to *P.neostrigosus* in appearance and has been treated as the same species (e.g. Pegler 1983; Li and Bau 2014; Li et al. 2015; Vargas-Isla et al. 2015). However, in our conception, these two species should be considered as two independent species. *Panusrudis* differed from *P.neostrigosus* in morphology by shorter metuloids and wider cheilocystidia. Moreover, the phylogenetic analysis results also support this conclusion.

### ﻿Key to the reported species of *Panus* from China

**Table:** 

1	Hymenial cystidia present and conspicuous, either with refractive gloeocystidia or thick-walled and metuloidal	**2**
–	Gloeocystidia and metuloids absent, sometimes small skeletocystidia present	**5**
2	Pileus shortly villous to hispid-strigose	**3**
–	Pileus glabrous, glabrescent or with a few fibrillose squamules	**4**
3	Cheilocystidia wide > 6 μm, metuloids projecting up to 15 μm beyond the basidia	***P.rudis***
–	Cheilocystidia wide < 6 μm, metuloids projecting up to 35 μm beyond the basidia	***P.neostrigosus***
4	Elongate fusoid gloeocystidis present	***P.strigellus***
–	Clavate to lageniform metuloidal cystidia present	***P.conchatus***
5	Lamellae is equal	***P.setiger***
–	Lamellae is unequal	**6**
6	Lamellae with cross-veins	**7**
–	Lamellae without cross-veins	**8**
7	With lamellulae of two lengths	***P.minisporus***
–	With lamellulae of six lengths	***P.baishanzuensis***
8	Pileus neither strongly striate nor plicate-sulcate, at times finely striate on weathered specimens	***P.velutinus***
–	Pileus radially striate, plicate-sulcate or with concentric zoning, glabrescent	**9**
9	Lamellae densely crowded; pileus finely hispid, radially striate but not sulcate	***P.ciliatus***
–	Lamellae moderately crowded: pileus almost glabrous, strongly plicate sulcate, stipe often very long	***P.similis***

## ﻿Discussion

In this study, 31 specimens of *Panus* from China were carefully examined. Through the combination of morphological and phylogenetic studies, two new species, *P.minisporus* and *P.baishanzuensis* and one new record from Zhejiang Province, China, viz. *P.similis*, have been discovered and described in detail, which increased the species diversity of *Panus* and expanded the distribution range of *P.similis*. According to Pegler, species belonging to *P.similis* complex were usually found near the Equator in Africa, South America, Australasia and Southeast Asia etc. (Pegler 1983). Additionally, there are also a few records in south China and southwest China (Li and Bau 2014). In contrast, *P.baishanzuensis* is located in east China, which greatly increases the distribution range of species in the *P.similis* complex.

There are quite a few species that are confusing and extremely similar in appearance. Species belonging to sect. Velutini sensu Pegler, such as *L.ciliatus*, *L.similis*, *L.hookerianus*, *L.tephroleucus*, *L.velutinus* and *L.fasciatus* belong to the *Lentinus* at first, because of their velutinate to strigose basidiomes and thick-walled skeletocystidia (Pegler 1983). However, with the changes in genus conceptions (Corner 1981; May and Wood 1995) and the consistency of phylogenetic and ontogenic studies (Hibbett and Vilgalys 1991; Hibbett et al. 1993), as a result, four species were combined as *Panus*, viz. *P.ciliatus*, *P.similis*, *Panushookerianus* (Berk.) T.W. May & A.E. Wood and *Panustephroleucus* (Mont.) T.W. May & A.E. Wood, which have a dimitic hyphal system consisting of thick-walled skeletal hyphae and generative hyphae (May and Wood 1995) and leaving *L.velutinus* and *L.fasciatus* within *Lentinus*. According to phylogenetic analysis, *L.fasciatus* and *P.ciliatus* are clustered with *P.rudis* and far away from *L.velutinus* (Douanla-Meli and Langer 2010), which is also consistent with the morphological characters. In the light of Douanla-Meli and Langer (2010), species of the *P.similis* complex usually have slender stipe (which can be up to twice as long as the diameter of the pileus). At the same time, the stipes of *L.fasciatus* and *P.ciliatus* are only sometimes slender, but usually short and stocky, which coincides with the stipe characteristics of *P.rudis* (Pegler 1983; Douanla-Meli and Langer 2010; Li and Bau 2014). Similar results were also obtained in this study.

Furthermore, some species within this complex were full of arguments. *Lentinusvelutinus* was proposed by Fries (1830); later, it was combined into *P.velutinus* (Fries 1838). Meanwhile, Berkeley (1843) described a new species, *Lentinusfulvus* Berk. Then, Pegler and Rayner (1969) transferred *L.fulvus* into *Panusfulvus* (Berk.) Pegler & R.W. Rayner. Afterwards, Corner described three varieties of *P.fulvus* from Malaysia, based on the pileus structure and dense lamellae (Corner 1981), which, together with *P.velutinus*, were rehabilitated by Pegler (1983) as *L.velutinus*. However, according to morphological studies (Pegler 1972, 1983; Corner 1981), *L.velutinus* does not have the skeleto-ligative hyphae typical of *Lentinus*, but has the skeletal hyphae typical of *Panus*, which coincides with the phylogenetic analysis (Douanla-Meli and Langer 2010; Luangharn et al. 2019). Thus, *L.velutinus* is more closely related to *Panus* than to *Lentinus*. *Lentinusfasciatus* was another controversial species; it was treated as a member of *Panus* in Singer and Pegler’s conception (Singer 1962; Pegler 1965). Later, the combination was rejected by Pegler (1983). However, the presence of skeletal hyphae and phylogenetic studies indicate a close genetic relationship with *Panus* (Pegler 1983; Douanla-Meli and Langer 2010).

In addition, some taxonomic problems exist in researching *Panus*. Firstly, synonyms led to confusion in species identification, such as *P.ciliatus* sensu May & Wood and *Panusbrunneipes* Corner. According to the description given by Corner (1981) for *P.brunneipes* and Pegler (1983) for *P.ciliatus* (= *L.ciliatus*), all these collections refer to the same species. However, based on the legitimate name, both names are accepted by Index Fungorum (http://www.indexfungorum.org) at the same time. However, the epithet ‘*ciliatus*’ is retained as the oldest one and has priority over ‘*brunneipes*’. Secondly, the boundary between *Pleurotus* and *Panus* needs to be refined. Species belonging to *Panus* are known to have a dimitic hyphal system with skeletal hyphae (Corner 1981; Pegler 1983). However, *Panusgiganteus* (Berk.) Corner and *Panustuber-regium* (Fr.) Corner, which also have skeletal hyphal, were transferred into *Pleurotus* (Singer 1951; Karunarathna et al. 2012). Phylogenetic analysis, based on ITS fragments, showed that they are closely related to species of Pleurotussubg.Coremiopleurotus (Klomklung et al. 2012; Karunarathna et al. 2016). It is worth mentioning that almost all of them have skeletal hyphae. Based on phylogenetic analyses (Stajic et al. 2005; Menolli et al. 2014; Shnyreva and Shnyreva 2015), species of the monomitic hyphal system and species of the dimitic hyphal system were clustered into a single unit, respectively. Obviously, these species are intermediate between *Panus* and the monomitic hyphal system species in *Pleurotus* (Fig. 1). Perhaps these species are attributed to a new genus-level unit that could serve as a boundary to distinguish between *Panus* and the remaining monomitic hyphal system species in *Pleurotus.* Thirdly, some species were always problematic for identification, for example, *P.neostrigosus* and *P.rudis* (Li and Bau 2014; Li et al. 2015). There are obvious differences between these two species. The metuloids of *P.neostrigosus* project up to 35 μm longer than *P.rudis* and the width of cheilocystidia is thinner than the latter. Thus, it is possible to distinguish these confusing species by combing the two characteristics (Pegler 1983; Douanla-Meli and Langer 2010; Li and Bau 2014; Luangharn et al. 2019). In addition, *P.neostrigosus* is clearly separate from *P.rudis* through phylogenetic analysis (Douanla-Meli and Langer 2010; Luangharn et al. 2019). Last, but not least, taxonomic research of the genus *Panus* is not evenly developed in China. The taxonomic research relating to *Panus* is mainly focused on northeast, southwest and south China. Additionally, the resources of *Panus* are waiting to be employed, especially in east and northwest China.

To address these issues, a more comprehensive study of *Panus* species that integrates morphological and systematic approaches to elucidate the relationships between different species is necessary. Reviewing the type specimens of every species in *Panus* is an essential task. At the same time, more gene fragments need to be obtained to construct more objective phylogenetic trees. With these preliminary preparations, the evolutionary relationship between the Chinese *Panus* data and the world’s species will be the subject of the next study.

## Supplementary Material

XML Treatment for Panus minisporus

XML Treatment for Panus baishanzuensis

XML Treatment for Panus similis

XML Treatment for Panus conchatus

XML Treatment for Panus neostrigosus

XML Treatment for Panus rudis

## ﻿Additional information

### Author ORCIDs

Jiajun Hu https://orcid.org/0000-0002-7562-7612

### Data availability

All of the data that support the findings of this study are available in the main text or Supplementary Information.

## Supplementary materials

Supplementary material 1 Newly obtained sequences Data type txt Explanation note ere are the ITS, nLSU, and *tef-1*α sequences obtained in this study. https://binary.pensoft.net/file/1060268This dataset is made available under the Open Database License (http://opendatacommons.org/licenses/odbl/1.0/). The Open Database License (ODbL) is a license agreement intended to allow users to freely share, modify, and use this Dataset while maintaining this same freedom for others, provided that the original source and author(s) are credited.Author: Lei Yue
